# Die psychotherapeutische Sprechstunde – Potenzial für eine Steuerungs- und Lotsenfunktion? Standardisierte Befragung von Psychotherapeut:innen im Kontext des Projektes Eva PT-RL

**DOI:** 10.1055/a-2694-5507

**Published:** 2025-10-21

**Authors:** Sandra Werner, Sarah Schlierenkamp, Pauline zur Nieden, Luisa Friedrich, Carina Abels, Klemens Höfer, Anke Walendzik, Dieter Best, Barbara Lubisch, Gebhard Hentschel, Daniel Thumm, Dagmar Wieczorek, Ursula Marschall, Bettina Meisel, Christa Schaff, Helene Timmermann, Jürgen Wasem, Silke Neusser

**Affiliations:** 1 Versorgungsforschung, Essener Forschungsinstitut für Medizinmanagement GmbH, Essen; 2 Wirtschaftswissenschaften, Universität Duisburg-Essen Lehrstuhl für Medizinmanagement, Essen; 3 Psychotherapie, Deutsche Psychotherapeutenvereinigung (DPtV), Berlin; 4 Ambulante Versorgung, AOK Bundesverband, Berlin; 5 Ambulante Versorgung & Vertragsstrategie, BARMER, Wuppertal; 6 Medizin/Versorgungsforschung, BARMER Institut für Gesundheitssystemforschung, Wuppertal; 7 Psychotherapie, Vereinigung für analytische und tiefenpsychologisch fundierte Kinder- und Jugendlichen-Psychotherapie in Deutschland e.V. Berlin; 8 Berufsverband für Kinder- und Jugendpsychiatrie, Psychosomatik und Psychotherapie in Deutschland e. V. (bkjpp), Mainz

**Keywords:** ambulante psychotherapeutische Versorgung, Reform der Psychotherapie-Richtlinie, psychotherapeutische Sprechstunde, Standardisierte Befragung, outpatient psychotherapeutic care, quantitative survey, reform outpatient psychotherapy, mental health

## Abstract

**Ziel:**

Untersucht wurde, ob die im Jahr 2017 in die ambulante psychotherapeutische Versorgung eingeführte psychotherapeutische Sprechstunde Potenzial für eine Steuerungs-/Lotsenfunktion (SLF) für Menschen mit psychischen Problemen hat und welche Faktoren die Umsetzung fördern bzw. hemmen können.

**Methodik:**

Zwischen 07/2021 und 03/2022 wurde eine Querschnittsbefragung einer geschichteten Zufallsstichprobe von 3.400 PT durchgeführt und deskriptiv ausgewertet.

**Ergebnisse:**

Für die Auswertung wurden 760 Fragebögen berücksichtigt. Die Mehrheit der PT stimmte zu, dass die Sprechstunde Beratung und Diagnostik ermögliche. Eine SLF der Sprechstunde wurde von 16% gesehen. Einen verbesserten Austausch mit anderen Fachdisziplinen oder Förderung sektorenübergreifender Kooperationen erkannten 5% der PT.

**Schlussfolgerung:**

Die Befragung gibt Hinweise darauf, dass eine verbesserte Vernetzung und Kommunikation unter Leistungserbringenden helfen könnten, um die Sprechstunde für eine SLF für Menschen mit psychischen Problemen zu nutzen.

## Abstract (en)

**Objective:**

To determine if the new care element of psychotherapeutic consultation (ptc), which was introduced in outpatient psychotherapeutic care in 2017, has the potential of coordinating care for people with mental health problems and to identify potential barriers and facilitators.

**Methods:**

A cross-sectional survey of a stratified random sample of 3,400 psychotherapists (pt) was conducted between 07/2021 and 03/2022. Descriptive analyses were performed.

**Results:**

760 questionnaires were included in the analysis. Most pt attributed appropriate advice or diagnostics to ptc. 16% of pt recognised that ptc had a role in coordinating care. 5% of pt recognised better communication with other specialist disciplines or improved cross-sectoral collaboration.

**Conclusion:**

The survey suggests that improved cooperation and communication between service providers may benefit the ptc for a potential role of coordinating the care of people with mental health problems.

## Einleitung

Das Hilfe- und Gesundheitsversorgungssystem für Menschen mit psychischen Erkrankungen in Deutschland ist vielfältig. Je nach klinischen Faktoren, Alter, Symptomschwere, Erkrankungsverlauf und Patientenpräferenz stehen niedrigschwellige psychosoziale Interventionen, medikamentöse und/oder psychotherapeutische Behandlungsalternativen sowie weitere Therapieverfahren zur Verfügung 1 . Ein wichtiges Element ist die ambulante vertragspsychotherapeutische Versorgung, die durch die Psychotherapie-Richtlinie (PT-RL) geregelt wird 2 . Weiterhin sind im Kontext des SGB V bspw. (teil-)stationäre oder tagesklinische Maßnahmen möglich. Darüber hinaus existieren auch SGB-V-übergreifende Angebote, wie durch die Kommunen finanzierte sozialpsychiatrische Dienste, Erziehungs-/Beratungsstellen oder Rehabilitationsmaßnahmen für chronisch psychisch kranke Menschen im Rahmen der öffentlichen Fürsorge oder nach Gesamt- und Teilhabeverfahren gemäß SGB IX und SGB XII 1 .

Um die Flexibilität der psychotherapeutischen Versorgung zu erhöhen, den Zugang zu verbessern und Wartezeiten zu reduzieren, wurde mit dem Gesetz zur Stärkung der Versorgung in der gesetzlichen Krankenversicherung eine Strukturreform der PT-RL angestoßen 3 . Diese trat im Jahr 2017 in Kraft und im Zuge dessen wurde u. a. die psychotherapeutische Sprechstunde (im Folgenden Sprechstunde) eingeführt 3 . Ziel der Sprechstunde war ein niedrigschwelliger zeitnaher (Erst-)Zugang in das ambulante psychotherapeutische System. Die Sprechstunde ist eine Pflichtleistung für niedergelassene Psychotherapeut:innen (PT) und ist für die meisten Patient:innen Voraussetzung für weitere psychotherapeutische Leistungen. Die Sprechstunde kann in Einheiten von mindestens 25 Minuten bis zu sechs Mal bei Erwachsenen je Krankheitsfall erbracht werden, bei Kindern und Jugendlichen bis zu 10-mal, ggf. unter Einbezug der Sorgeberechtigten 2 . PT mit vollem Versorgungsauftrag müssen mindestens 100 Minuten pro Woche für Sprechstunden bereitstellen 2 .

In den Sprechstunden kann ein Erstgespräch zur Abklärung des Vorliegens einer psychischen behandlungsbedürftigen Erkrankung erfolgen und es besteht die Möglichkeit eine (differential-)diagnostische Abklärung und Beratung zu möglichen, dem individuellen Behandlungsbedarf entsprechenden Versorgungsangeboten vorzunehmen 2 3 . Die Sprechstunde dient somit der Beratung und Information von Patient:innen. Überdies umfasst sie die Möglichkeit zu Prävention, einer ersten Diagnosestellung sowie einer dem Bedarf entsprechenden Empfehlung für eine fachspezifische Maßnahme im Leistungsbereich des SGB V (z. B. ambulante Richtlinientherapie (RL-T), stationäre Versorgung) oder darüber hinaus (u. a. Selbsthilfegruppen/Beratungsstellen). Am Ende der Sprechstunde wird eine Patienteninformation (PTV 11) ausgehändigt, die schriftlich festhält, ob weitere Maßnahmen erfolgen sollten und wenn ja, welche.

In den Diskussionen vor Einführung der Sprechstunde gab es den Wunsch, dass der Sprechstunde eine Steuerungs- und Lotsenfunktion für Menschen mit psychischen Problemen zukommen könnte, um das komplexe und fragmentierte Hilfe- und Versorgungssystem für psychische Erkrankungen angemessen und effizient einzusetzen 4 5 . Insbesondere Menschen, bei denen innerhalb der Sprechstunde kein Bedarf für Leistungen gemäß SGB V identifiziert wird, könnten unterstützt werden, für sie passende Angebote zu ermitteln.

Im Gutachten des Sachverständigenrats aus dem Jahr 2018 wird als Ziel der Steuerung von Gesundheitsdienstleistungen das Patientenwohl und eine bedarfsgerechte, Patientenpräferenzen berücksichtigende Versorgung verstanden, bei der dem aufgeklärten, mündigen Betroffenen eine Hilfestellung zur Orientierung im Versorgungssystem gegeben wird 5 . In Bezug auf die Sprechstunde ließe sich eine Steuerungs- bzw. Lotsenfunktion als eine Form der bedarfsgerechten Patientennavigation verstehen. Dieses Verständnis von Steuerungs- bzw. Lotsenfunktion ist zu unterscheiden vom Lotsenansatz, bei dem Patient:innen über den gesamten Behandlungsverlauf einer Erkrankung begleitet werden oder deren gesamte Behandlung koordiniert wird, wie bspw. durch Hausärzt:innen (HÄ) im Rahmen der hausarztzentrierten Versorgung.

Die bei Einführung der Sprechstunde geführte Diskussion zeigt, dass der Sprechstunde neben der Möglichkeit eines niedrigschwelligen Zugangs auch Potenzial zugeschrieben wurde, sowohl die interdisziplinäre/-professionelle und sektorenübergreifende Versorgung von Menschen mit psychischen Problemen zu stärken als auch SGB V-übergreifende Leistungen bzw. regionale Versorgungsangebote im Kontext psychischer Erkrankungen koordinierter einzubinden 4 5 . Eine solche Vernetzung könnte Menschen mit psychischen Erkrankungen zu einer besseren Orientierung im Hilfe- und Versorgungssystem und einer selbstbestimmteren Versorgung verhelfen. Zudem sollen durch diagnostische Abklärungen im Rahmen der Sprechstunde die entsprechenden Bedarfe frühzeitig erkannt und eine adäquate Versorgung unter zielgerichtetem Einsatz therapeutischer Maßnahmen ermöglicht werden 3 . Mit einer bedarfsorientierten Steuerung ist die Erwartung einer verbesserten Versorgungsqualität sowie einer effizienteren Ressourcennutzung verbunden, welche durch eine stärkere Vernetzung der verschiedenen Leistungsangebote und gesteigerte Kommunikation zwischen Akteur:innen in der Versorgung psychisch kranker Menschen gefördert würde. Da die verschiedenen Leistungsstrukturen finanziell unabhängig voneinander sind, bestehen bisher nur unzureichende Anreize, die Vernetzung zum Wohle der Patient:innen zu stärken 1 .

Mit der vorliegenden Untersuchung soll der Frage nachgegangen werden, inwieweit Potenzial hinsichtlich der mit der Einführung der Sprechstunde diskutierten Steuerungs- bzw. Lotsenfunktion aus Sicht von PT in der Versorgung Erwachsener (PTE) sowie Kinder und Jugendlicher (PTKJ) bereits zu erkennen ist. Zudem werden weitere Faktoren wie Vernetzung und Kommunikation unter Leistungserbringenden untersucht, die die Umsetzung einer möglichen Steuerungs- bzw. Lotsenfunktion im Kontext der Sprechstunde im Praxisalltag fördern bzw. hemmen können. Diese Untersuchung stellt einen Teilaspekt des Projekts Eva PT-RL (Evaluation der Reform der PT-RL; Förderkennzeichen: 01VSF19006) dar 6 .

## Methodik

Datengrundlage ist eine Querschnittsbefragung von PT. Aus dem Adresspool eines Zielgruppenspezialisten wurde jeweils eine hinsichtlich des Ausbildungshintergrunds geschichtete Zufallsstichprobe von 1.700 PTE bzw. PTKJ gezogen, die an der vertragsärztlichen bzw. -psychotherapeutischen Versorgung teilnehmen. Hierbei wurde vorgegeben, dass jeweils 80% (n=1.360) einen psychologischen Hintergrund haben sollten und 20% (n=340) einen ärztlichen, etwa Fachärzt:innen (FA) für Psychosomatische Medizin und Psychotherapie, FA für Psychiatrie und Psychotherapie, FA für Psychotherapie oder FA für Kinder-/Jugendpsychiatrie und -psychotherapie. Die Gesamtstichprobe umfasste 34.151 Psychotherapeut:innen, 5.270 niedergelassene Ärzt:innen der Fachrichtung Psychiatrie (mit Zusatz Psychotherapie) und 3.253 niedergelassene Ärzt:innen der Fachrichtung Psychosomatische Medizin und Psychotherapie. Die Stichprobe wurde im Juli 2021 postalisch angeschrieben und im Oktober (PTKJ) bzw. November (PTE) 2021 erfolgte ein Reminderschreiben. Mit dem Fragebogen wurde ein Informations- und Aufklärungsschreiben zur Erhebung verschickt. Die Studie wurde von der Ethik-Kommission der Medizinischen Fakultät der Universität Duisburg-Essen (20-9792-BO) beraten und zustimmend bewertet.

Aufgrund des geringen Rücklaufs der PTKJ, rekrutierten im Januar 2022 die im Projekt Eva PT-RL beteiligten Berufsverbände per Mail unter ihren Mitgliedern weitere PTKJ. Um eine Online-Teilnahme zu ermöglichen, wurde der Erhebungsbogen in digitaler Form mittels LimeSurvey erstellt. Die Befragung wurde im Dezember 2021 (PTE) bzw. März 2022 (PTKJ) beendet.

### Fragebogenentwicklung

Eine orientierende Literaturrecherche zur Umsetzung der PT-RL ergab nur wenige inhaltlich ähnlich ausgerichtete empirische Studien. Diese fanden bei der Erstellung des Fragebogens Berücksichtigung. Zur weiteren Exploration von Fragestellungen im Kontext der Strukturreform der PT-RL wurden sechs Fokusgruppen und fünf Einzelinterviews mit PT, Kostenträger:innen und Patientenvertreter:innen durchgeführt 7 . Auf dieser Basis wurden für die standardisierte Befragung geschlossene Fragen zu folgenden Oberthemen entwickelt: telefonische Erreichbarkeit, Terminservicestelle, Sprechstunde, psychotherapeutische Akutbehandlung, RL-T, Rezidivprophylaxe, Praxisorganisation. Ziel war es, Erkenntnisse zur Umsetzung der Reform der PT-RL, zur Zufriedenheit der PTE und PTKJ mit der Reform sowie zu möglichen Problemen der Umsetzung zu gewinnen. Der Fragebogen umfasste 29 themenspezifischen Fragen. Zudem wurden zur Einordnung der Studienpopulation Fragen zu soziodemographischen Merkmalen, Zugehörigkeit zur Kassenärztlichen Vereinigung (KV) und Ausbildungshintergrund gestellt. Für das vorliegende Manuskript wurden Einschätzungen der PT zum Potenzial der Sprechstunde hinsichtlich einer dem Bedarf angepassten Steuerungs- bzw. Lotsenfunktion, (Differential-)diagnostik, inner-/intersektoralen Kommunikation und Beratung anhand einer 5-stufigen Likert-Skala erhoben ( Tab. 2 ). Zudem wurde eine Frage zu den empfohlenen Maßnahmen im Rahmen einer Sprechstunde gestellt. Hierbei wurden Antwortoptionen gemäß PTV 11 vorgegeben und die Befragten gebeten, anzugeben, wie häufig (in%) sie diese empfehlen.

**Table:** **Tab. 2** Einschätzungen zur Sprechstunde.

Die Einführung der Sprechstunde…	PTE		PTKJ	
	**n**	Zustimmung (%) Teils, teils (%) Ablehnung (%)*	**n**	Zustimmung (%) Teils, teils (%) Ablehnung (%)*
**…hat die gewünschte Lotsenfunktion (=bedarfsgerechte Steuerung der Patient:innen) erfüllt.**	**441**	17	**356**	16
		33		36
		51		48
**…hat dazu geführt, dass Patient:innen bedarfsorientiert versorgt werden.**	**439**	7	**355**	17
		30		31
		63		52
**…ermöglicht es, Patient:innen angemessen zu beraten.**	**441**	39	**356**	46
		38		34
		23		20
**…hat zu einer besseren Zusammenarbeit mit psychosozialen Angeboten geführt.**	**441**	4	**356**	6
		16		25
		80		69
**…hat zu einem besseren Austausch mit Haus-/Fachärzt:innen geführt.**	**441**	2	**356**	4
		7		17
		91		79
**…hat zu einer besseren sektorenübergreifenden Kooperation geführt.**	**440**	4	**358**	5
		11		16
		86		79
**…kann zu gezielter (Differential-)Diagnostik genutzt werden.**	**440**	46	**356**	48
		29		29
		26		23
**…führt zu einem Druck, Vorgaben umzusetzen, obwohl keine Therapieplätze vorhanden sind.**	**440**	78	**353**	69
		14		18
		8		13
**…wird von Patient:innen gut angenommen.**	**439**	57	**357**	60
		33		31
		10		9

PTE: Psychotherapeut:in für Erwachsene; PTKJ: Psychotherapeut:in für Kinder/Jugendliche. *Für eine bessere Lesbarkeit wurden die 5-stufige-Likert-Skala (stimme voll zu, stimme zu, teils, teils, stimme nicht zu, stimme gar nicht zu) zu 3 Kategorien zusammengefasst.

Die Fragebögen für PTE und PTKJ unterschieden sich in sechs Items. PTKJ wurden bspw. zusätzlich befragt, wie Sorgeberechtigte/Bezugspersonen in die psychotherapeutische Versorgung eingebunden werden.

Die Fragebogenentwicklung erfolgte in Abstimmung mit allen Projektpartner:innen. Als Pre-Test wurden die Fragebögen mit Vertreter:innen der entsprechenden Professionen diskutiert und im Anschluss entsprechend den Rückmeldungen angepasst.

### Auswertung

In die Auswertung wurden Fragebögen von PT mit KV-Zugehörigkeit einbezogen, die über Angaben zur Person hinausgehende Antworten beinhalteten. Keiner der Befragten beantwortete alle Fragen (Items). Aufgrund dessen wurde für jedes Item die Gesamtzahl der gültigen Antworten (inkl. Prozentangaben) ausgewiesen. Aufgrund des explorativen Charakters der Befragung, erfolgte eine deskriptive Auswertung mittels uni-/bivariater Analysen. Je nach Variablentyp wurden Häufigkeitsverteilungen und Lageparameter ermittelt.

## Ergebnisse

### Studienpopulation

Insgesamt gingen 760 Fragebögen online und schriftlich ein. Nach Eingang der Erhebungsbögen erfolgte die Zuordnung der Fragebögen zur Gruppe der PTE oder PTKJ. Eine Einbeziehung in die Stichprobe beider Gruppen erfolgte, wenn im schriftlich erhobenen Fragebogen angegeben wurde, dass Erwachsene und KJ versorgt wurden.

Von den Befragten PTE antworteten 16 (4%) online und 433 (96%) schriftlich [PTKJ: 195 (54%) bzw. 167 (46%)]. In beiden Gruppen konnten jeweils zwei Fragebögen aufgrund der Angabe, keiner KV anzugehören, nicht eingeschlossen werden, sodass 447 (PTE) bzw. 360 Fragebögen (PTKJ) zur Auswertung vorlagen. Somit lag die Rücklaufquote bei den PTE bei 26,3%. Eine Rücklaufquote für die PTKJ konnte nicht ermittelt werden, da nicht ausgeschlossen werden kann, dass es Überschneidungen in den kontaktierten Stichproben gab.

Von den Befragten waren 68% (PTE) bzw. 62% (PTKJ) über 50 Jahre alt und gut drei Viertel waren weiblich. In beiden Gruppen praktizierte etwa ein Viertel der Befragten in einem Ort mit weniger als 20.000 Einwohner:innen und etwa die Hälfte in einem Ort mit mehr als 100.000 Einwohner:innen.

Mehrheitlich hatten die PTE ein Psychologiestudium absolviert (89%) und über die Hälfte (57%) besaß eine Zulassung für verhaltenstherapeutischen Verfahren. Bei den PTKJ absolvierte gut die Hälfte ein pädagogisches und 21% ein psychologisches Studium. Mit 53% hatten mehr als die Hälfte der PTKJ eine Zulassung für das analytische und tiefenpsychologische Verfahren ( Tab. 1 ).

**Table:** **Tab. 1** Charakteristika der Studienpopulation.

		PTE		PTKJ	
Merkmal	Ausprägung	n	%	n	%
**Altersgruppe**	21–40 ^a^	42	10	39	11
	41–50	100	23	99	28
	51–60	155	35	122	34
	>60	146	33	99	28
	**Gesamt**	**443**	**100**	**359**	**100**
**Geschlecht**	Männlich	98	23	82	24
	Weiblich	331	77	261	76
	**Gesamt**	**429**	**100**	**343**	**100**
**Absolvierter Studiengang**	Medizin	41	9	45	13
	Psychologie	389	89	71	21
	Pädagogisch	/	/	187	54
	Anderes	6	1	42 ^b^	12
	**Gesamt**	**436**	**100**	**345**	**100**
**Psychotherapeutische Verfahren**	Nur tiefenpsychologisch	111	26	63	18
	Verhaltenstherapie	250	57	79	23
	Analytisch und tiefenpsychologisch	57	13	185	53
	Sonstige	18	4	24	7
	**Gesamt**	**436**	**100**	**351**	**100**
**Größe des Ortes, in dem praktiziert wird**	Landgemeinde (<5.000 EW)	35	8	19	5
	Kleinstadt (5.000 bis<20.000 EW)	69	16	74	21
	Mittelstadt (20.000–100.000 EW)	110	25	96	27
	Großstadt (>100.000 EW)	227	52	171	48
	**Gesamt**	**441**	**100**	**360**	**100**

EW: Einwohner; PTE: Psychotherapeut:in für Erwachsene; PTKJ: Psychotherapeut:in für Kinder/Jugendliche; ^a^ Zusammenfassung der Gruppen 21–30/31–40 aufgrund geringer Fallzahlen. ^b^ z.B. pädagogische Studiengänge kombiniert mit Kunst-/Musiktherapie oder Medizin/Psychologie, Grundschullehramt, etc. .

### Erfahrungen mit der Sprechstunde

Von den Befragten stimmten 39% (PTE) bzw. 46% (PTKJ) der Aussage zu, dass die Sprechstunde eine angemessene Beratung von Patient:innen ermögliche und 46% (PTE) und 48% (PTKJ) stimmten zu, dass die Sprechstunde zu gezielter (Differential-)diagnostik genutzt werden kann. Der Aussage, dass die Sprechstunde eine Lotsenfunktion erfülle, stimmten 17% (PTE) bzw. 16% (PTKJ) zu, etwa ein Drittel der Befragten war indifferent. Eine Zustimmung, dass die Einführung der Sprechstunde zu einer bedarfsorientierten Versorgung der Patient:innen führe, gaben 7% (PTE) bzw. 17% (PTKJ), wohingegen 63% (PTE) bzw. 52% (PTKJ) dieser Aussage nicht zustimmten.

Hinsichtlich des Aspekts des Austauschs mit anderen Akteur:innen stimmten nur wenige Befragte zu, dass die Einführung der Sprechstunde 1. zu einer besseren Zusammenarbeit mit psychosozialen Angeboten (PTE: 4%; PTKJ: 6%), 2. einem besseren Austausch mit Haus-/Fachärzt:innen (PTE: 2%; PTKJ: 4%) oder 3. einer besseren sektorenübergreifenden Kooperation geführt hat (PTE: 4%; PTKJ: 5%).

Darüber hinaus gaben 78% (PTE) bzw. 69% (PTKJ) an, dass mit der Einführung der Sprechstunde ein Druck entstehe, Vorgaben umzusetzen, obwohl keine Therapieplätze vorhanden sind. Gleichzeitig wird die Sprechstunde nach Einschätzung der befragten PT von Patient:innen (bzw. Bezugspersonen) gut angenommen ( Tab. 2 ).

Anhand der Angaben zur Häufigkeit empfohlener Maßnahmen im Anschluss an eine Sprechstunde gemäß PTV 11 zeigt sich, dass sowohl PTE als auch PTKJ in nur wenigen Fällen nach einer Sprechstunde keine Maßnahmen oder Präventionsmaßnahmen empfehlen (s. e-supplement). Vielmehr gaben 60% der befragten PTE an, dass sie häufig eine Verhaltenstherapie empfehlen und 48% der PTKJ empfehlen häufig eine tiefenpsychologisch fundierte Therapie. Weiterhin werden in etwas geringerem Maße fach-/hausärztliche Abklärungen empfohlen, seltener stationäre oder rehabilitative Maßnahmen. Angebote außerhalb des SGB V, wie (Erziehungs-)Beratungsstellen oder Selbsthilfegruppen, werden vor allem durch PTKJ empfohlen.

Mehr als ein Drittel der Befragten gab an, dass sie nicht wissen, ob ihre Vermittlung erfolgreich ist, wenn eine Weiterbehandlung bei ihnen selbst nicht möglich ist. Von den PT, die eine Info erhalten, gaben ca. 30% an, dass eine Weitervermittlung nicht gelinge, weil es zu wenig Kapazitäten im Umkreis gibt bzw. es gelingt eine Weitervermittlung mit Wartezeit (PTE: 15%; PTKJ: 29%). Nur 2% (PTE) bzw. 4% (PTKJ) der Befragten gaben an, dass Weitervermittlungen ohne große Verzögerungen gelingen. Der Hauptgrund für die Verweisung von Patient:innen im Anschluss an eine Sprechstunde sind fehlende Kapazitäten ( Tab. 3 ).

**Table:** **Tab. 3** Angaben zur Weitervermittlung nach Sprechstunde.

Merkmal	Ausprägung	PTE		PTKJ	
		n	%	n	%
Gründe für die Verweisung von Patient:innen nach Beendigung der Sprechstunde*	Keine Kapazitäten	356	81	285	80
	Anderes Therapieverfahren angemessen	334	76	231	65
	Persönliche Gründe der Patient:innen	68	16	78	22
	Problematische Therapeut-Patient:innen-Passung	234	53	140	39
	Terminfindung schwierig	203	46	153	43
	Anfahrt der Patient:innen zu lang	108	25	154	43
	Kommt nicht vor	15	3	17	5
	**Gesamt**	**440**	**100**	**356**	**100**
Unterstützung von Patient:innen, die im Anschluss an eine Sprechstunde für eine Akutbehandlung/RL-T weitervermittelt werden müssen*	Hinweis auf Möglichkeit der Terminvereinbarung über Terminservicestelle	313	72	210	59
	Ausgabe einer Liste mit PT	212	49	235	66
	Kontaktherstellung zu PT innerhalb des eigenen Netzwerkes	167	38	186	52
	Hinweis auf Krankenkasse als Unterstützer	202	46	150	42
	Gar nicht. Keine Kapazität, Patient:innen bei der PT-Suche zu unterstützen	57	13	25	7
	Gar nicht. Nicht meine Aufgabe, Patient:innen bei der PT-Suche zu unterstützen	26	6	7	2
	**Gesamt**	**435**	**100**	**355**	**100**
**Gelingt es, Patient:innen weiterzuvermitteln, die nach einer Sprechstunde nicht in Ihrer Praxis weiter behandelt (d. h. Akutbehandlung/RL-T) werden können?**	Nein, nicht genügend Kapazitäten im Umkreis.	108	32	85	28
	Ja, ohne große Verzögerungen. Das psychotherapeutische Angebot im Umkreis ist gut.	3	1	9	3
	Ja, ohne große Verzögerungen. Weitervermittlung über persönliches Netzwerk.	6	2	11	4
	Ja, mit Wartezeiten.	51	15	87	29
	Ich weiß nicht. Ich gebe Patient:innen PT-Kontaktdaten, erhalte aber keine Rückmeldung, ob es zu einer erfolgreichen Weitervermittlung gekommen ist.	133	39	109	36
	Ich weiß nicht. Ich vermittle keine Patient:innen weiter.	38	11	4	1
	**Gesamt**	**339**	**100**	**305**	**100**

^*^ Mehrfachnennungen möglich. RL-T: Richtlinientherapie; PT: Psychotherapeut:innen; PTE: Psychotherapeut:in für Erwachsene; PTKJ: Psychotherapeut:in für Kinder/Jugendliche.

## Diskussion

Die vorliegende Befragung niedergelassener PT in Deutschland zu Erfahrungen mit der neu eingeführten Sprechstunde zeigt, dass die bei Einführung formulierten Ziele einer gezielten (Differential-)Diagnostik als auch einer angemessenen Beratung nach Einschätzung der Befragten erreicht wurden und Patient:innen die Sprechstunde gut annehmen. Allerdings gibt sie auch Hinweise darauf, dass das Potenzial der Sprechstunde nicht voll ausgeschöpft ist. So geben die Befragten an, dass sich die Kommunikation und Vernetzung mit anderen Akteur:innen des Hilfe- und Versorgungssystems aufgrund der Einführung der Sprechstunde nicht wesentlich verändert hat. Auch einen Einfluss auf eine bedarfsgerechte Steuerung bzw. Versorgung wird nur von wenigen Befragten erkannt.

Vielmehr wird die Sprechstunde eher als ein der Therapie vorgelagerter Baustein wahrgenommen, als ein vernetzendes oder steuerndes Element, welches interdisziplinär, sektoren- und SGB-V-übergreifend genutzt werden könnte. Dabei stößt die Verpflichtung der Durchführung der Sprechstunde vor allen Dingen bei einem Mangel an Therapieplätzen bei der Weitervermittlung an Grenzen. Die Möglichkeit, die Sprechstunde als ein Instrument zu nutzen, dass durch Kommunikation und Vernetzungen innerhalb des komplexen Hilfe- und Versorgungssystems für psychische Erkrankungen eine bedarfsgerechte Versorgung fördern könnte, scheint bisher von wenigen Befragten genutzt zu werden. Dieses kann darin begründet sein, dass eine solche Funktion bei Einführung der Sprechstunde nicht explizit formuliert wurde 2 3 . Demnach könnten die Befragten es auch nicht als ihre Aufgabe sehen, eine Vernetzung und Kommunikation im Sinne einer verbesserten Patientensteuerung aktiv zu verbessern.

Auch die Umsetzbarkeit von empfohlenen Maßnahmen mittels PTV 11 könnte von den PT mitgedacht werden. Diese steht in Abhängigkeit von bestehenden Kapazitäten der PT aber auch individuellen Ressourcen der Patient:innen. Zudem wurde die Einstellung der Befragten zu einer Steuerungs- bzw. Lotsenfunktion und ob sie sich dazu befähigt sähen, nicht abgefragt, was die Einordnung der Ergebnisse erschwert. Zudem ist es möglich, dass der in der Befragung genutzte Begriff der bedarfsgerechten Steuerung individuell unterschiedlich interpretiert wurde, wobei der Pretest diesbezüglich keine Unklarheiten ergab. Anhand des normativen Konzepts zur Bedarfsgerechtigkeit zeigt sich jedoch, dass der Begriff schwierig allgemeingültig zu definieren ist. So ist diesem zufolge eine Versorgung bedarfsgerecht, wenn sie in qualitativer und quantitativer Hinsicht dem Bedarf der Patient:innen entspricht 8 . Dabei wird zwischen dem objektiven Bedarf, der sich bspw. an der Krankheitsschwere orientieren kann, und dem subjektiven Bedarf, d. h. den individuellen Präferenzen und Wünschen der Patient:innen, unterschieden 8 . Hingegen wird im Kontext der Gesundheitssystemforschung ein versorgungsbedürftiger Bedarf im gesetzlichen Sinn durch Ärzt:innen bzw. PT bestimmt 9 .

Die Evaluation der Funktion der Sprechstunde des Unterausschusses des G-BA kommt auf Basis der Auswertung von KBV-Daten zu dem Schluss, dass die Steuerungsfunktion der Sprechstunde erfüllt sei 10 . Dabei wird eine Steuerungsfunktion aus Sicht der Autor:innen dahingehend erkannt, dass 40% der Versicherten, die eine Sprechstunde wahrgenommen haben, im Anschluss keine weiteren psychotherapeutischen Leistungen erhalten und somit die Sprechstunde in dem Sinne steuere, diejenigen zu selektieren, die eine bzw. keine ambulante psychotherapeutische Versorgung benötigen 10 . Hingegen weisen die Ergebnisse der vorliegenden Befragung darauf hin, dass eher selten keine Maßnahmen im Anschluss an eine Sprechstunde empfohlen werden und auch andere Maßnahmen als psychotherapeutische Leistungen empfohlen werden. Dieses deckt sich mit Angaben von Versicherten der BARMER, wonach knapp 10% der Befragten angaben, dass ihnen keine Maßnahme empfohlen wurden 11 . Unklar ist weiterhin, welche Maßnahmen im Anschluss an eine Sprechstunde empfohlen und umgesetzt werden, wenn es keine psychotherapeutischen Leistungen sind. Daneben ist es möglich, dass ein gewisser Teil andere Formen der Versorgung oder Unterstützung benötigt. Es besteht somit weiterhin Forschungsbedarf zu der Frage, inwieweit eine bedarfsgerechte Steuerung unter Einbindung des gesamten Hilfe- und Versorgungssystems für psychische Erkrankungen sowie insbesondere auch präventive Maßnahmen mit Hilfe der Sprechstunde möglich ist.

Kapazitätsmängel, Probleme in der Weitervermittlung, ein Mangel an inner-/intersektoralem Austausch, unterschiedlich finanzierte Leistungsstrukturen sowie ein Mangel an Kenntnissen des gesamten Hilfe- und Versorgungssystems könnten mögliche Hemmschwellen für die Umsetzung einer Steuerungs- bzw. Lotsenfunktion darstellen und eine Begründung für die Vielzahl an ablehnenden Antworten hinsichtlich der Erfüllung einer Lotsenfunktion durch die Sprechstunde darstellen.

### Limitationen

Bei der vorliegenden Befragung handelt es sich um eine Querschnittsbefragung. Eine weitere Limitation ist die zusätzliche Rekrutierung der PTKJ über die Berufsverbände, da es sich aufgrund dieses Vorgehens nicht mehr um eine Zufallsstichprobe handelt. Dieses könnte zu einer Einschränkung der Übertragbarkeit der Ergebnisse führen. Allerdings deckt sich die Studienpopulation der vorliegenden Untersuchung weitgehend hinsichtlich der Parameter Alter und Geschlecht mit der deutschlandweiten Verteilung aller niedergelassenen PT 12 . Zudem kann nicht ausgeschlossen werden, dass die Teilnahmebereitschaft unter PT, die besonders unzufrieden sind, höher ist als unter PT, die zufrieden sind. Allerdings deutet die Verteilung über die Antwortkategorien nicht auf ein homogenes Antwortverhalten hin.

### Mögliche Ansätze zur Weiterentwicklung der psychotherapeutischen Versorgung

Eine Vielzahl an Modellen und Verträgen einzelner Krankenkassen auf Länderebene oder regional begrenzt, die u. a. durch bessere Vernetzung von Leistungserbringenden positive Effekte für die Versorgung von Menschen mit psychischen Erkrankungen erzielen 1 , unterstreichen die Relevanz einer koordinierten Versorgung. Auch in den Ergebnissen des vom Innovationsfonds geförderten Projekts eines gestuften und koordinierten Versorgungsmodells für bestimmte psychische und neurologische Erkrankungen (NPPV) wird dargestellt, dass für knapp 50% der teilnehmenden Leistungserbringenden eine Vernetzung untereinander als auch die Steuerung der Patientenversorgung als sehr wichtig wahrgenommen wird 13 . Die vorliegende Befragung von PT zu Erfahrungen mit der Sprechstunde deutet darauf hin, dass auf verschiedenen Ebenen Ansätze möglich wären, um die Funktion und das Potenzial der Sprechstunde hinsichtlich einer Steuerungs- bzw. Lotsenfunktion im Hilfe- und Versorgungssystem psychischer Erkrankungen und entsprechender Vernetzung zu stärken. Ein Ansatzpunkt zur Verbesserung der Versorgungsqualität durch eine interdisziplinäre und sektorenübergreifende Versorgung und Vernetzung könnte in einer verstärkten und standardisierten Kommunikation liegen. Dazu ließen sich neben dem bestehenden PTV 11, das als Informations- und Kommunikationsbasis zwischen Leistungserbringenden für einen fallbezogenen Austausch genutzt werden kann, bspw. Fallkonferenzen oder Qualitätszirkel nutzen. Zudem müssten Kompetenzen der Steuerung- und Lotsenfunktion klar zugeordnet und definiert werden und in den entsprechenden Aus-, Fort- und Weiterbildungscurricula eingebunden werden. Als weiteren Ansatzpunkt ließe sich diskutieren, inwieweit die Kenntnisse über das gesamte System und insbesondere zu regionalen Angeboten verbessert werden könnten.

Grundsätzlich bedarf es eines ganzheitlichen koordinierten Ansatzes, wenn mit der Sprechstunde eine bedarfsgerechte Steuerungs- bzw. Lotsenfunktion über Disziplin-, Sektoren- und SGB-Grenzen hinaus in einem hochkomplexen und fragmentierten System wie der Versorgung von psychisch kranken Menschen gewünscht ist 1 . Auch vor dem Hintergrund, dass psychische Erkrankungen oftmals im Zusammenhang mit somatischen Erkrankungen auftreten, ist eine gute Vernetzung von Leistungserbringenden und eine koordinierte Versorgung wichtig 14 .

Die vorliegende Befragung gibt Hinweise darauf, an welchen Stellen Potenzial für einen verbesserten Einsatz der Sprechstunde gegeben ist, um die bedarfsgerechte Versorgung von Menschen mit psychischen Erkrankungen flächendeckend zu verbessern und knappe Ressourcen adäquat und effizient zu nutzen.

## Fördermittel

Innovationsausschuss des Gemeinsamen Bundesausschusses — Förderkennzeichen: 01VSF19006

## Zusätzliches Material

Zusätzliches Material Zusätzliches Material
